# A Novel Approach of Optimum Time Interval Estimation for Al-7.5Si/Al-18Si Liquid–Liquid Bimetal Casting in Sand and Metallic Moulds

**DOI:** 10.3390/ma16083004

**Published:** 2023-04-10

**Authors:** Naglaa Fathy, Mohamed Ramadan, Khalid M. Hafez, Fahad Abdulaziz, Badreddine Ayadi, Abdulaziz S. Alghamdi

**Affiliations:** 1 Department of Physics, College of Science, University of Ha’il, Hail P.O. Box 2440, Saudi Arabia; 2 College of Engineering, University of Ha’il, Hail P.O. Box 2440, Saudi Arabiaa.alghamdi@uoh.edu.sa (A.S.A.); 3 Central Metallurgical Research and Development Institute (CMRDI), P.O. Box 87, Helwan 11421, Egypt; 4 Department of Chemistry, College of Science, University of Ha’il, Hail P.O. Box 2440, Saudi Arabia; fah.alanazi@uoh.edu.sa; 5 Laboratory of Applied Fluid Mechanics, Environment and Process Engineering ”LR11ES57”, National School of Engineers of Sfax (ENIS), University of Sfax, Route Soukra Km 3.5, Sfax 3038, Tunisia

**Keywords:** time interval, liquid–liquid, bimetal casting, sand, metallic, moulds

## Abstract

This work describes a novel approach for Al-7.5Si/Al-18Si liquid–liquid bimetal casting in sand and metallic moulds. The aim of the work is to facilitate and develop a simple procedure to produce an Al-7.5Si/Al-18Si bimetallic material with a smooth gradient interface structure. The procedure involves the theoretical calculation of total solidification time (TST) of the first liquid metal (M1), pouring the liquid metal (M1), and allowing it to solidify; then, before complete solidification, the second liquid metal (M2) is introduced into the mould. This novel approach has been proven to produce Al-7.5Si/Al-18Si bimetal materials using liquid–liquid casting. The optimum time interval of Al-7.5Si/Al-18Si bimetal casting with modulus of cast M_c_ ≤ 1 was estimated based on subtracting 5–15 s or 1–5 s from TST of M1 for sand and metallic moulds, respectively. Future work will involve determining the appropriate time interval range for castings having modulus ≥ 1 using the current approach.

## 1. Introduction

There is an urgent need to produce materials that can withstand the harsh conditions of chemical corrosion, mechanical erosion, and work at high temperatures. Sometimes, engineering products need to work in different media; for example, one of the sides of a product may be subjected to mechanical friction while the other side of the same piece is subjected to high impact stress. Bimetallic composite materials are the most suitable materials for these different impact conditions. Bimetallic materials are composed of two different layers that differ in terms of their microstructure and their physical, chemical, and mechanical properties [1,2,3,4,5].

Bimetallic materials are produced in many manufacturing processes, including welding, rolling, and casting [6,7,8,9,10]. The metal casting method is the most common and widely used method in the production of bimetallic materials. Casting of bimetallic materials is divided into two main types, namely, liquid–solid methods and liquid–liquid methods [11]. Each of these two methods of casting bimetallic materials has its own advantages and difficulties. In the liquid–solid method, one metal is melted in a heating furnace, then this molten metal is poured into the cavity of the mold containing the solid metal. One of the advantages of this methods is that it requires only one furnace to melt and cast a single metal. The difficulty with this method is that the surface of the solid metal needs to be prepared right away to ensure that the molten metal can be poured on top of it and form a strong bond after the liquid metal solidifies [12,13,14,15,16,17,18,19,20,21].

In the liquid–liquid method, two different metals are melted in two different furnaces, then the first metal is poured, then after a while the other metal is poured [22,23]. Obtaining a strong bond between the two metals is one of this method’s benefits. However, there are challenges, as there must be two gating systems and two melting furnaces [12,13,14,15,16,17,18,19,20,21]. Research has previously focused on using liquid–solid methods to produce bimetallic composites due to ease of implementation and the requirement for only one melting furnace to melt one metal. On the other hand, liquid–liquid methods have seen very little research published due to the requirement for two melting furnaces and two gating systems. In addition, it is essential to cautiously control the time between pouring the first metal and the second metal [24].

Aluminum silicon alloys are used to fabricate bimetallic composite material using the cast-decant-cast process [25]. In the decanting fabrication method, the first alloy is poured into the mold, then when a specific thickness is solidified, the remaining part of liquid is decanted. Only then is the second alloy poured into the mold. For fabrication of Al–21Si/Al–7.5Si bimetallic material [22], the liquid–liquid method was used to produce a high-quality Al/Al bimetallic material. By using different time intervals, a bimetallic material was successfully fabricated using a 10 s time interval. Extensive experiments on the thermal process window for Cu–Sn alloy/Cu by static liquid–liquid bimetal casting was conducted in [26]. It was reported in [26] that a cohesive bond between Cu-Sn alloy and pure Cu partners was achieved by forming a solid interface solution. Previous studies [27] have shown that liquid–liquid bimetal casting of carbon steel/chromium cast-iron bimetal can be used to fabricate a distinguishable composite interlayer. Previous studies [28] have emphasized good interface bonding between Zn alloy and an Al alloy matrix using a novel liquid–liquid lost-foam casting with the assistance of a solid Al interlayer.

Thus far, high varieties of bimetallic composites have been manufactured via the liquid–solid compound casting method. However, there are limited publications that mention using liquid–liquid compound casting to process bimetallic composites. Additionally, specifying the optimal time interval estimation for liquid–liquid bimetal casting is difficult. This paper addresses a novel approach to time interval estimation for Al/Al liquid–liquid bimetal casting in sand and metallic moulds. In addition, we demonstrate that the optimal time interval can be calculated using the total solidification time of the first metal poured in the liquid–liquid bimetal casting process.

## 2. Experimental

### 2.1. Martials

Al pure metal, 6061Al alloy, recycling Al-Si alloy (from used pistons), and Al-25% Si master alloy were used to prepare Al-7.5Si (M1) and Al-18Si (M2) alloys. A heat resistance muffle furnace was used to melt a charge of 1.2 kg of alloy constituents in a graphite crucible. The chemical compositions of the Al alloys are shown in Table 1.

### 2.2. Bimetal Casting Process

**Figure 1:**
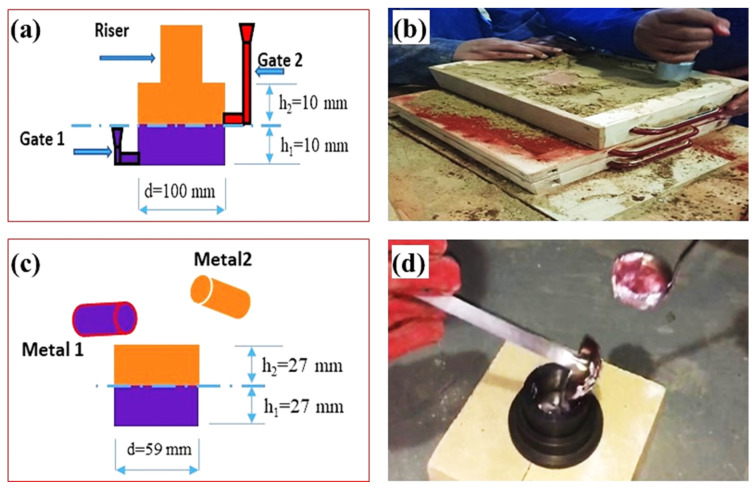
Gating and risering systems (**a**), sand molding of bimetal sand casting (**b**), metallic mould top pouring (**c**), and double pouring of metallic mould casting (**d**), the two colors represent M1 and M2.

The M1 and M2 alloys bars were melted in two separate graphite crucibles using a heat resistance muffle furnace. Argon gas was used as a protective gas for the melting of both M1 M2. The Al Alloys were heated to a temperature of 750 °C and held for 30 min. After holding, the M1 and M2 melts were skimmed of preformed dross. Subsequently, the M1 and M2 melts with a temperature of 720 °C were poured one after another into the sand mould (for group specimen A) and metallic moulds (for group specimen B). The pouring system and a double pouring of sand and metallic mould casting of the liquid–liquid Al/Al bimetal-casting setup used in the current work are displayed in Figure 1.

For the preparation of sand moulds, about 8% of the bentonite binder and about 3% water were added to the silica sand and it was mixed well for about 30 min. The metallic mould was turned from carbon steel with a cylindrical inner diameter of 59 mm and an outer diameter of 75 mm. The percentage of volume for liquid M1 to compared to liquid M2 in this bimetal casting was 1:1. First, for bimetal sand mould casting, liquid M1 was poured at a temperature of 720 °C into the sand mould cavity. Second, after a certain interval time (60–120 s), liquid M2 was poured into the mold above the M1. For bimetal metallic mould casting, liquid M1 was poured at a temperature of 720 °C into the preheated (270 °C) metallic mould cavity. Then, after a certain interval time (10–30 s), liquid M2 was poured into the mold above the M1.

### 2.3. Bimetal Casting Characterization

Specimens were longitudinally cut, ground, polished, and etched with 0.5% HF + 99.5% H_2_O solution. An optical microscope connected to an advanced digital camera was used for microstructure observations. A scanning electron microscope (SEM) was used for observation of the microstructure interface. The chemical compositions of the interface were characterized by EDS analyses.

### 2.4. Total Solidification Time Estimation

In the current research, the total solidification time (TST) of M1 was used as an important tool for estimating the optimum time interval between pouring M1 and M2. In liquid–liquid bimetal casting, a smooth interfacial bonding layer free of both voids and unbonded areas is the main challenge for obtaining high quality bimetal castings. TST was calculated using Chvorinov’s rule; see Equations (1) and (2). Casting metal volume (V_c_), cooling surface area (A_c_), and superheating (ΔTs) effects were considered for TST modification according to previous reports [29,30]. The constant n (power of modulus of cast (M_c_ = V_c_/A_c_)) was estimated to be 2 for a cast with M_c_ ≥ 1 and equal to 1 for a small cast with M_c_ ≤ 1, according to previous reports [29,30]. All of the definitions and thermophysical property values in Equations (1) and (2) are shown in Table 2.

(1) $$TST=B{\left(\frac{V_{C}}{A_{C}}\right)}^{n}$$

(2) $$B={\left[\frac{\rho_{m}L}{\left(T_{m}-T_{o}\right)}\right]}^{2}\left[\frac{\pi }{4k\rho c}\right]\left[1+{\left(\frac{c_{m\;}\Delta T_{s}}{L}\right)}^{2}\right]$$

## 3. Results and Discussion

**Figure 2:**
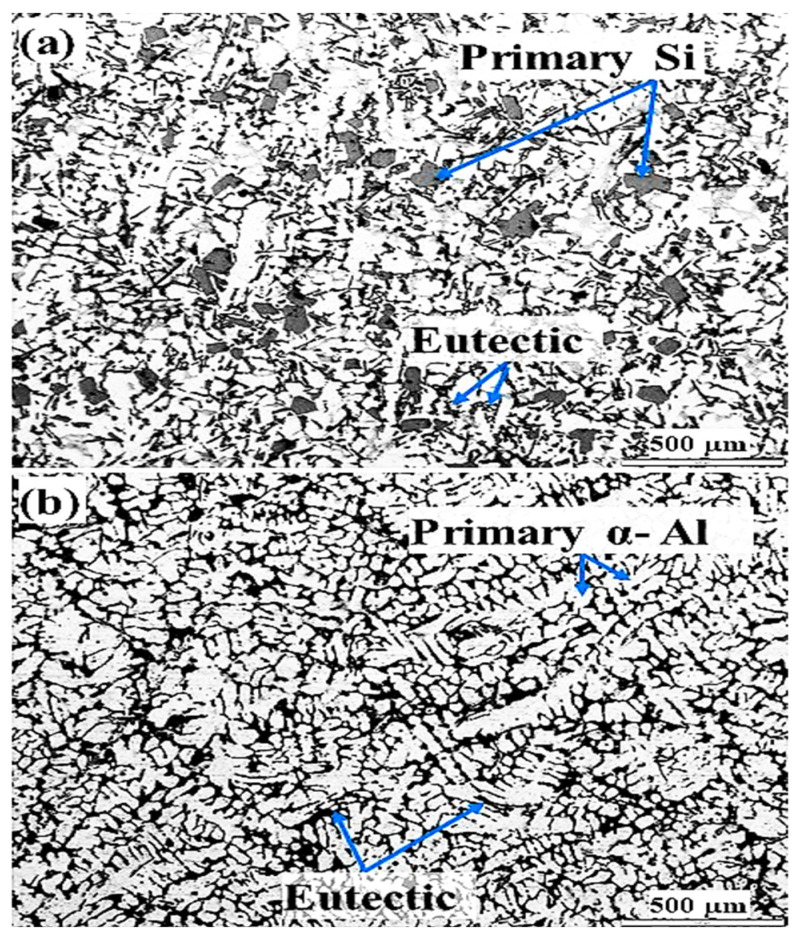
Microstructure of (**a**) Al-18Si and (**b**) Al-7.5Si aluminum alloys.

The microstructures of M2 (Al-18Si) and M1 (Al-7.5Si) are shown in Figure 2. The primary Si and eutectic are clearly observed in microstructure of M2 (Figure 2a). Otherwise, primary α-Al and eutectic are observed in the microstructure of M1 (Figure 2b). It is commonly known [37] that adding Si to pure Al significantly enhances the casting characteristics of aluminum alloys. Al-Si alloys’ fluidity, hot tear resistance, and other casting properties are enhanced by adding up to about 25% Si. A reduction in coefficient of thermal expansion and specific gravity are reported with additions of Si to Al alloys. Based on the relation between fluidity and cooling rate, the optimum range of Si content in Al-Si alloys can be assigned to metal casting processes, whereas a lower Si content (Si ≤ 7) is recommended for slow cooling rate sand casting and a higher Si content (Si ≥ 7) for gravity permanent mold casting [37].

### 3.1. Interfacial Microstructure in Sand Mould Al/Al Bimetal Casting

**Figure 3:**
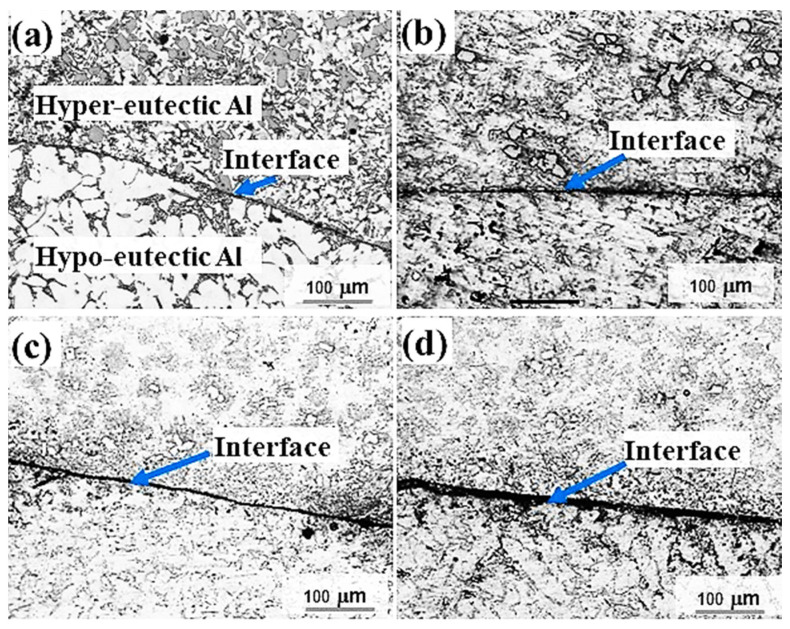
Interfacial microstructure of Al/Al bimetal casting in sand mould for four different time intervals: (**a**) 60 s, (**b**) 75 s, (**c**) 90, and (**d**) 120 s.

The interfacial microstructures of Al/Al bimetal castings in the sand mould for four different time intervals (60 s to 120 s) are shown in Figure 3. The interfacial microstructure of Al/Al sand mould bimetal castings using a time interval of 60 s shows a relatively adherent bond structure (Figure 3a). When increasing the time interval above 60 s to 75 s and above, the interfacial microstructures show higher percentages of unbonded areas, eventually reaching complete bimetal separation when using a time interval of 120 s. Aluminum alloys oxidize easily in molten states to provide a continuous self-limiting film. The increase in the melting temperature as well as the presence of reactive elements in Al alloys such as Na, Mg, and Ti increases the oxidation rate of aluminum alloys [38]. The formation of oxide films on the surface as well as the outer surface solidified layer thicknesses of M1 increases when increasing the total solidification time. In sand moulding, the cooling rate is relatively low, which resulted in increasing the oxide surface film and outer surface solidified layer thicknesses in both metal/sand interface and the metal/air interface on the top of the cast surface.

Generally, in bimetal casting, especially during pouring of the second molten metal onto the first, the induced turbulence during pouring results in breaking the preformed oxides/solidified shell layer of the first poured metal, leading to entrainment of oxide particles in the bimetal interfacial area and the second metal. In liquid–liquid bimetal sand casting, the oxides formed by direct oxidation in air at the top surface of the outer solidified shell of the first poured metal (M1) become stronger and more difficult to break with increasing solidification time. Increasing oxides/outer solidified shell of the first poured metal (M1) suppressed formation of the hypo/hyper Al-Si interlayer (Figure 3b–d).

### 3.2. Interfacial Microstructure in Metallic Mould Al/Al Bimetal Casting

**Figure 4:**
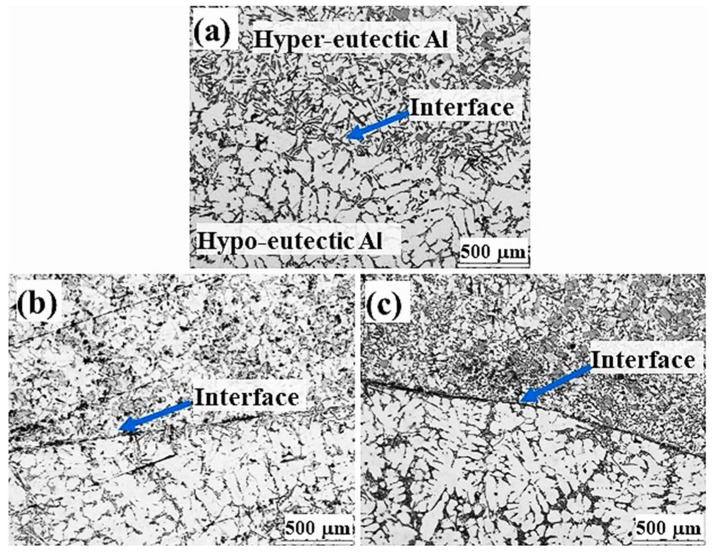
Microstructure of Al/Al bimetal casting in metallic mould casting for three different time intervals: (**a**) 10 s, (**b**) 20 s, and (**c**) 30 s.

The microstructures of Al/Al bimetal casting in metallic mould casting for three different time intervals (10 s, 20 s and 30 s) are shown in Figure 4. The Al-7.5Si hypoeutectic structures seen at the bottom of Figure 4a–c show a primary α-Al phase and a eutectic phase, whereas the Al-18Si hypereutectic alloys show a primary Si phase and a eutectic phase, as shown at the top of Figure 4a–c. For Al/Al bimetal casting in the metallic mould using a 10 s time interval, an interfacial layer of eutectic structure containing an angular eutectic aluminum and fine eutectic silicon phase can be seen in Figure 4a. However, for Al/Al bimetal casting using 20 s and 30 s time intervals, thin interfacial layers containing unbonded areas can be observed (Figure 4b,c).

**Figure 5:**
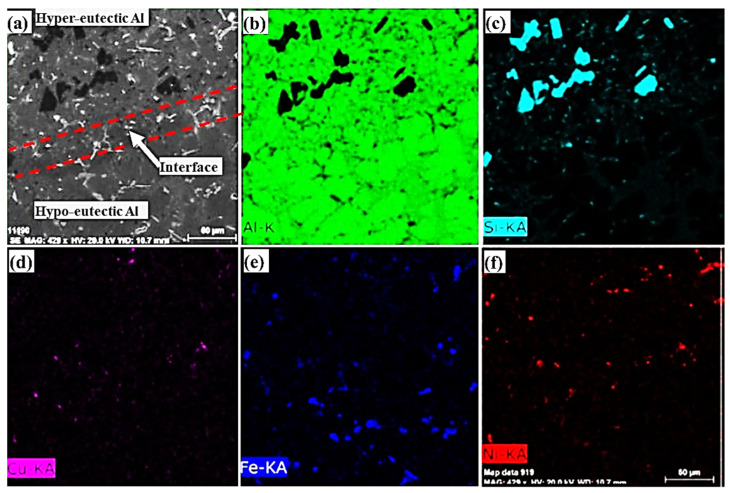
(**a**) SEM micrograph and concentration mappings of (**b**) Al, (**c**) Si, (**d**) Cu, (**e**) Fe, and (**f**) Ni for the Al/Al bimetal casting in the metallic mould using a 10 s time interval.

SEM micrograph and element concentration mappings of the Al/Al bimetal casting in the metallic mould using a 10 s time interval are shown in Figure 5. The element concentration mappings of the Al/Al bimetal casting in the metallic mould confirm the presence of the Al-7.5Si hypoeutectic structure and Al-18Si hypereutectic structure, as well as the eutectic interfacial layer structure. Fabrication of Al/Al bimetal using a liquid–liquid method with a 10 s time interval shows the best interfacial bonding structure in the metallic mould.

**Figure 6:**
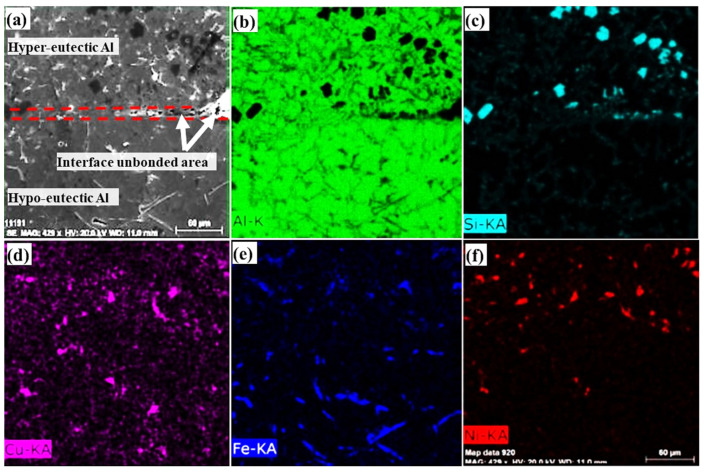
(**a**) SEM micrograph and concentration mappings of (**b**) Al, (**c**) Si, (**d**) Cu, (**e**) Fe, and (**f**) Ni for the Al/Al bimetal casting in the metallic mould using a 20 s time interval.

Unbonded interfacial areas are clearly observed when using higher time intervals of 20 and 30 s (Figure 4b,c). As mentioned before, in Al/Al bimetal casting increasing the time interval increases both oxides and solidifies the surface layer thicknesses of M1, prohibiting the smooth formation of the eutectic interlayer. SEM micrographs and element concentration mappings of the Al/Al bimetal casting in the metallic mould using a 20 s time interval are shown in Figure 6. The element concentration mappings for the Al/Al bimetal casting in metallic mould confirm the presence of unbonded areas in the interfacial layer structure.

Based on the microstructure and SEM results and comparing the sand mould and metallic mould for Al/Al bimetal liquid–liquid casting, the optimal time interval using sand moulding is 60 s or less and the optimal time interval using the metallic mould is 10 s. The relatively lower cooling rate in the sand mould increases the total solidification time of first poured metal (M1), which leads to an increase in the time interval needed in the sand mould in order to achieve an adherent and thin interfacial layer with Al/Al bimetal casting. It is worth noting here that in order to obtain the appropriate time interval to fabricate Al/Al bimetal using the liquid–liquid method in a sand or metallic mould it is necessary to conduct many costly laboratory experiments.

### 3.3. Time Interval Estimation for Al/Al Bimetal Casting in Sand and Metallic Moulds

In the current work, the optimal time interval between pouring M1 and pouring M2 in the sand and metallic moulds was estimated based on the calculation of TST for M1. Accordingly, the time interval between pouring M1 and M2 could be optimized. To ensure the formation of an optimal interfacial layer in bimetal casting, the optimal time interval should be slightly less than the TST of M1.

**Figure 7:**
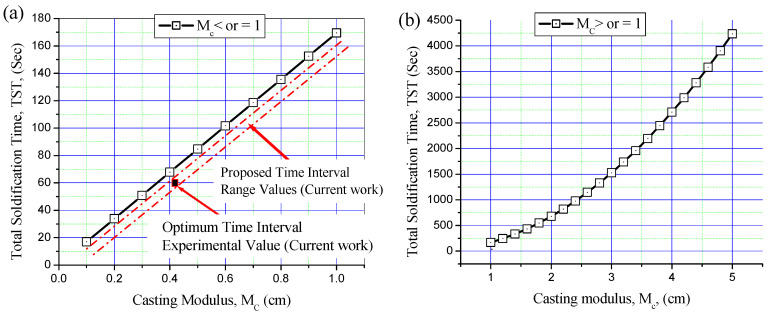
Total solidification time (TST) of Al-7.5Si alloy in sand mould for castings with different casting moduli: (**a**) M_c_ ≤ 1 and (**b**) M_c_ ≥ 1. Areas between the two red dotted lines represent the proposed time interval range values.

**Figure 8:**
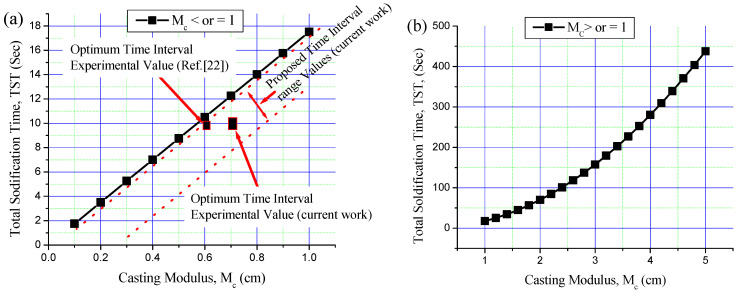
Total solidification time (TST) of Al-7.5Si alloy in metallic mould for castings with different casting moduli: (**a**) M_c_ ≤ 1 and (**b**) M_c_ ≥ 1. Areas between the two red dotted lines represent the proposed time interval range values.

Based on Chvorinov’s rule in Equations (1) and (2), as well as on the thermophysical properties of Al-7.5Si aluminum alloy and of the sand and metallic moulds, the TST of the first poured metal (M1) was calculated for small castings (M_c_ ≤ 1) and large castings (M_c_ ≥ 1) in the sand and metallic moulds (Figure 7 and Figure 8). TST is mainly dependent on the volume of cast and cooling surface area and on the thermophysical properties of the poured metal and the mould. For the same poured metal and the same mould materials, TST increases with increasing modulus of the cast (M_c_).

The current casting moduli (M_c_) of M1 in the sand and metallic moulds were calculated. The Mc of 0.41 cm for the sand mould and 0.7 cm for the metallic mould were calculated and kept constant for all bimetal castings. Figure 7 shows the TST of Al-7.5Si alloy in the sand mould for castings with Mc ≤ 1 and Mc ≥ 1. The optimum experimental time interval of 60 s for Al/Al bimetal casting in the sand mould is shown in Figure 7a. The experimental time interval of 60 s is less than that obtained by theoretical TST of around 10 s for the same cast modulus. The optimal time interval of Al/Al bimetal casting in the sand mould was estimated by subtracting 5–15 s from the TST value of M1, considering theoretical and experimental errors. Figure 8 shows the TST of Al-7.5Si in the metallic mould for castings with Mc ≤ 1 and Mc ≥ 1. The optimum current experimental time interval of 10 s for Al/Al bimetal casting in the metallic mould is shown in Figure 8a. The experimental time interval of 10 s is less than that obtained by theoretical TST, with about 2 s for the same cast modulus. Previous work [24] reported an optimum experimental time interval of 10 s for Al-21Si/Al-7.5Si bimetal composite in a metallic mould. The optimum time interval of Al/Al bimetal casting in metallic moulding was estimated by subtracting 1–5 s from the TST value of M1, considering theoretical and experiment errors.

By using the current estimated time interval, it can be confirmed that the presence of a small portion of liquid on the surface or subsurface of facilitates the mixing of the new M2 alloy in the interfacial layer between the two alloys. For a casting modulus ≥ 1 of M1 in a sand or metallic mould, the time interval is believed to be lower than the TST, with a relatively higher subtracting value due to increasing oxides and solidification of the outer top surface layer with time. Definite range values of time intervals of Al-7.5Si/Al-18Si liquid–liquid bimetal casting in sand or metallic moulds of Mc ≥ 1 will estimated in a future work by the current authors. Other research avenues might extend the present research to study the strength of the connection between the two alloys. In addition, for Al-7.5Si/Al-18Si liquid–liquid bimetal casting in sand moulds with M_c_ ≥ 1, it is highly recommended that an appropriate flux be used to minimize preformed oxides on the surface of M1 before pouring M2 in order to improve the resulting bimetallic connections.

## 4. Conclusions

A novel approach to estimation of the optimal time interval for Al-7.5Si/Al-18Si liquid–liquid bimetal casting in sand and metallic moulds was developed in this study. An Al-7.5Si/Al-18Si bimetal casting was successfully fabricated in sand and metallic moulds using a liquid–liquid method with a 60 s and 10 s time interval, respectively. A clear interlayer with a eutectic structure for Al-7.5Si/Al-18Si bimetal casting fabricated in a metallic mould was achieved. In sand and metallic moulds, the liquid–liquid method with time intervals longer than 60 s and 10 s, respectively, showed unbonded areas due to the formation of oxides and solidification of the top surface layer. The optimal time intervals of Al-7.5Si/Al-18Si bimetal castings in sand and metallic moulds were estimated based on the TST of M1 by subtracting 5–15 s or 1–5 s, respectively, from the TST value of M1.

## Figures and Tables

**Table 1:** Chemical compositions of the materials (wt.%).

Materials	Chemical Compositions (wt.%)								
	Si	Cu	Mg	Ni	Fe	Zn	Ti	Mn	Al
Al-7.5Si (M1)	7.45	0.86	0.21	0.10	0.71	0.10	0.05	-	Bal.
Al-18Si (M2)	18.80	0.63	0.66	1.80	0.63	0.07	0.08	0.10	Bal.

**Table 2:** Thermophysical properties of Al-7.5Si aluminium alloy and sand, and metallic moulds.

	Sand Mould	Metallic Mould
Melting temperature of the liquid (K), T_m_	913	
Superheat (K), ΔT_s_	80	
Temperature of the mould (in K), T_o_	288	543
Latent heat of fusion (J kg^−1^), L	4.45 × 10^5^ [31]	
Thermal conductivity of the mould (Wm^−1^ K^−1^), k	1.4 [32]	19.54 [33,34]
Density of the mould (kg m^−3^), ρ	1570 [32]	7800 [33,34]
Specific heat of the mould (J kg^−1^ K^−1^), C	1153 [32]	522 [33,34]
Density of the metal (kg m^−3^), ρ_m_	2.76 × 10^3^ [35]	
Specific heat of the metal (J kg^−1^ K^−1^), C_m_	1058 [36]	

## Data Availability

Not applicable.
